# Characteristics of children with hip displacement in cerebral palsy

**DOI:** 10.1186/1471-2474-8-101

**Published:** 2007-10-26

**Authors:** Gunnar Hägglund, Henrik Lauge-Pedersen, Philippe Wagner

**Affiliations:** 1 Department of Orthopaedics, Lund University Hospital, S-221 85 Lund, Sweden; 2 Swedish National Competence Centre for Musculoskeletal Disorders, Lund University Hospital, S 221 85 Lund, Sweden

## Abstract

**Background:**

Hip dislocation in children with cerebral palsy (CP) is a common and severe problem. The dislocation can be avoided, by screening and preventive treatment of children with hips at risk. The aim of this study was to analyse the characteristics of children with CP who develop hip displacement, in order to optimise a hip surveillance programme.

**Methods:**

In a total population of children with CP a standardised clinical and radiological follow-up of the hips was carried out as a part of a hip prevention programme. The present study is based on 212 children followed until 9–16 years of age.

**Results:**

Of the 212 children, 38 (18%) developed displacement with Migration Percentage (MP) >40% and further 19 (9%) MP between 33 and 39%. Mean age at first registration of hip displacement was 4 years, but some hips showed MP > 40% already at two years of age. The passive range of hip motion at the time of first registration of hip displacement did not differ significantly from the findings in hips without displacement.

The risk of hip displacement varied according to CP-subtype, from 0% in children with pure ataxia to 79% in children with spastic tetraplegia. The risk of displacement (MP > 40%) was directly related to the level of gross motor function, classified according to the gross motor function classification system, GMFCS, from 0% in children in GMFCS level I to 64% in GMFCS level V.

**Conclusion:**

Hip displacement in CP often occurs already at 2–3 years of age. Range of motion is a poor indicator of hips at risk. Thus early identification and early radiographic examination of children at risk is of great importance. The risk of hip displacement varies according to both CP-subtype and GMFCS. It is sometimes not possible to determine subtype before 4 years of age, and at present several definitions and classification systems are used. GMFCS is valid and reliable from 2 years of age, and it is internationally accepted.

We recommend a hip surveillance programme for children with CP with radiographic examinations based on the child's age and GMFCS level.

## Background

Hip displacement in cerebral palsy (CP) is common. The risk of progression to hip dislocation is 15–20% in the total population of children with CP [1,2]. Hip dislocation in people with spasticity is a severe problem, with a high risk of pain, development of severe contractures, windswept deformity and scoliosis, resulting in problems with positioning, sitting, standing and walking [3-5].

Hip dislocation in cerebral palsy is preventable, by repeat radiographic and clinical examinations, and preventive treatment in hips with displacement of the femoral head [6-8]. To optimise the screening procedure it is important to know the characteristics of those children at risk of dislocation.

A CP register and a health care programme aimed at preventing hip dislocation and severe contractures was initiated in southern Sweden in 1994. We have used this total material to study the clinical manifestations in children who developed displacement related to those who did not.

## Methods

In 1994, a register and a health care programme for children with CP (CPUP) was started in southern Sweden [6,9]. The register includes all children with CP born after 1 January 1990, living in the area (the counties of Skåne and Blekinge) with a total population of about 1.3 million. The total population of children born 1990 and later was systematically reviewed in 1998 and 2002 in order to find all children with CP. The prevalence of CP in the study period was 2.4/1000 live births [10]. Since 2005 CPUP has been a national health care register approved by the National Board of Health and Welfare in Sweden. CPUP includes a continuing standardised follow-up of passive joint motion, gross and fine motor function, clinical findings and treatment. Only children who were alive at two years of age and had their brain lesion before that age were included. The CP-subtype was determined after the fourth birthday according to Hagberg et al [11]. The gross motor function was classified according to the age-related gross motor function classification system, GMFCS [12] (Table 1). The local physiotherapist and occupational therapist examine the children twice a year until the age of six years and then once a year. Measurement of passive joint of motion is done with a goniometer in stated and standardised positions and joint angles.

**Table 1:** Summary of the criteria for the Gross Motor Function Classification System(GMFCS) for children 6–12 years of age.

Level I	Walks without restrictions, limitations in more advanced gross motor skills.
Level II	Walks without restrictions, limitations walking outdoors and in the community.
Level III	Walks with assistive mobility devices, limitations walking outdoors and in community.
Level IV	Self-mobility with limitations, children are transported or use power mobility outdoors and in the community.
Level V	Self-mobility is severely limited, even with the use of assistive technology.

CPUP also includes a standardised radiographic follow-up of the children's hips. The hips were examined with anteroposterior radiograph at diagnosis, then at least once a year in children with diplegic, tetraplegic or dystonic type of CP until eight years of age, then individually (Table 2). Children with spastic hemiplegia or pure ataxia were examined radiographically at four years of age. If the radiograph was normal, no further examinations were undertaken, unless the clinical follow-up showed decreasing range of motion of the hips.

**Table 2:** The radiographic prevention programme 1994–2006

Spastic hemiplegia Ataxic CP	Radiographic examination at 4 years of age*
Spastic diplegia Spastic tetraplegia Dyskinetic CP Not classified CP	Radiographic examination at diagnosis, then at least once a year until 8 years of age, then individually.

*Further radiographic examination if MP > 33%, or a if a decreasing hip-ROM is revealed in the CPUP

**Figure 1:**
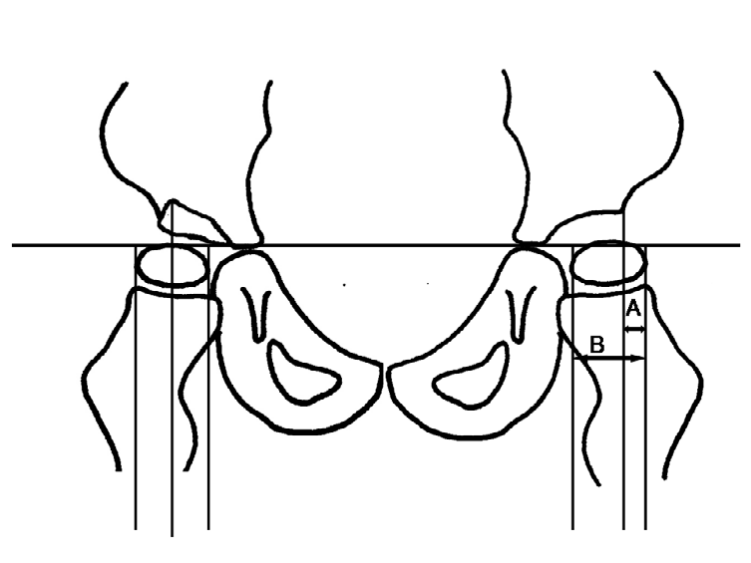
Measurement of Migration Percentage (MP). MP = A/B × 100. On the right hip with a "Gothic arch" formation of the lateral margin, the midpoint of the arch is used as reference point.

The migration percentage (MP) was measured on all radiographs (Figure 1). All measurements were performed by one of the authors. In hips with a "Gothic arch" formation of the lateral margin, the midpoint of the arch was used as reference point [13]. The hip programme started in 1994 and includes children born in 1992 or later. In the present study, children with hips showing MP > 33% and > 40% were analysed in relation to those with hips below these limits. The hip with highest MP determined the classification of the child.

In the present study the children born 1992–1997 have been analysed. Only those born in the area and those who have moved into the area before 2 years of age, and still living in the area at 9 years of age and participating in CPUP, were included in the study. We identified 212 children fulfilling the criteria for inclusion (Table 3). The distribution of subtypes and GMFCS-levels of the 8 children not participating in CPUP, and the 10 children not participating in the radiographic screening in CPUP is presented in Table 4. One child with dystonia and GMFCS level V, not participating in CPUP had developed bilateral hip dislocation. Most children not participating in the radiographic screening were at GMFCS level I. The systematic review of all children in 1998 and 2002 identified some children that were late participating in CPUP. For the analysis of age at lateral displacement and the analysis of range of motion (ROM) at time of lateral displacement, these four children were excluded (Table 3).

**Table 3:** The number of children with cerebral palsy in the population and in the study

Born in the area 1992–1997	**214**
Moved into the area before 2 years of age	**22**
Moved out of the area before 9 years of age	**3**
Died before 9 years of age	**3**
Not participating in CPUP	**8**
Not participating in radiographic hip screening	**10**
Number of children in the present study (diagnosis, GMFCS)	**212**
Late participation in CPUP	**4**
Number of children in the present study (age, ROM)	**208**

**Table 4:** Distribution of CP-subtype and GMFCS-level in 8 children not participating in CPUP and 10 children not participating in the radiographic hip screening in CPUP.

**CP-subtype/GMFCS-level**	**Not partici-pating in CPUP**	**Not partici-pating in radiographic hip screening**	**Total**
**Spastic**			
Hemiplegia		7	7
Tetraplegia	1		1
Diplegia	1	1	2
**Dyskinetic**			
Dystonia	1		1
Atetosis			
**Ataxic**	4	2	6
**Unclassified**	1		1
			
**GMFCS**			
I	3	9	12
II		1	1
III			
IV			
V	2		2
Unclassified	3		3

In the CPUP database all measurements of ROM are registered. Passive range of hip abduction, external rotation, internal rotation and extension was recorded from the examination performed at the same age as the first radiographic presentation of hip displacement. The Cox model [14] was used for evaluating effects of range of hip motion on the risk of developing MP > 33% or > 40%. Estimates of effects are presented as relative risk, both crude and adjusted for potential confounders (GMFCS level, CP-subtype, age). The estimates can be interpreted as the ratio of risk between two patient groups that differ by only one degree of ROM.

## Results

Of the 212 children, 38 (18%) developed MP > 40% and a further 19 (9%) developed MP 33–39%. The remaining 155 children (73%) did not show lateral displacement exceeding 33%. No child had developed a hip dislocation. None of the 155 children with MP < 33% had been operated with preventive surgery. Of the 57 children with MP > 33%, one was operated with femoral varisation osteotomy and three were operated with selective dorsal rhizotomy when MP was 33–39%. Three of these children remained with MP < 40%. One boy operated with selective dorsal rhizotomy at 6 years of age developed MP > 40% at twelve years of age.

**Figure 2:**
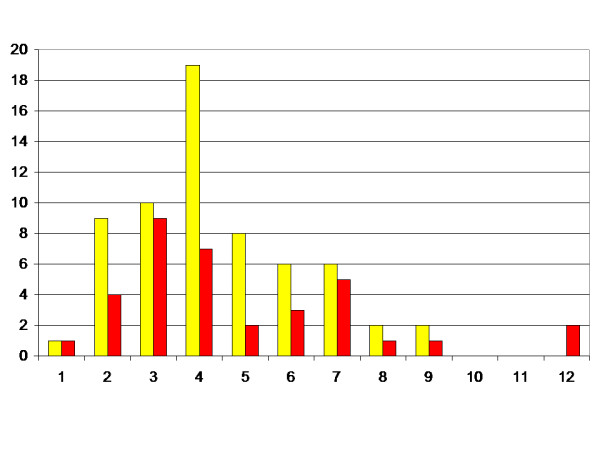
Number of children related to age (years) at first registration of MP above 33% (yellow) and 40% (red).

The age at first registration of MP > 33% or > 40% is presented in Figure 2. The children with MP > 40% are presented with both the age at registration of MP > 33%, and the age at registration of MP > 40%. Median age at first registration of MP > 33% was 4 years (range 1–9 years), and median age at first registration of MP > 40% was 4 years (range 1–12 years).

The range of hip motion at age at first registration of MP > 33% or > 40% is presented in Table 5. In the crude relative risk estimates a significant (p < 0.05) effect was found for external rotation and extension for MP > 33% and external rotation and abduction for MP > 40% (Table 6). In the adjusted estimates no change in risk related to hip ROM was found.

**Table 5:** Range of hip motion (median, quartiles) at time of first presentation of hip displacement (MP > 33% and 40%).

	MP > 33%	MP > 40%
Abduction	40 (30–41)	30 (25–40)
External rotation	50 (35–60)	45 (35–60)
Internal rotation	50 (45–65)	50 (44–65)
Extension	0 (0-0)	0 (0-0)

**Table 6:** Relative risk (with 95% CI) of developing MP > 33% and > 40%, related to range of hip motion. * = p < 0.05.

	Crude	Adjusted
MP > 33%		
Extension	0.93 (0.88–0.99)*	0.97 (0.92–1.03)
External rotation	1.03 (1.01–1.06)*	0.99 (0.97–1.02)
Internal rotation	1.02 (1.00–1.05)	1.02 (0.99–1.05)
Abduction	0.97 (0.94–1.00)	1.00 (0.96–1.03)
MP > 40%		
Extension	0.95 (0.89–1.02)	0.99 (0.92–1.05)
External rotation	1.04 (1.01–1.07)*	0.99 (0.96–1.02)
Internal rotation	1.01 (0.98–1.05)	1.02 (0.98–1.05)
Abduction	0.95 (0.91–0.97)*	0.97 (0.93–1.01)

**Figure 3:**
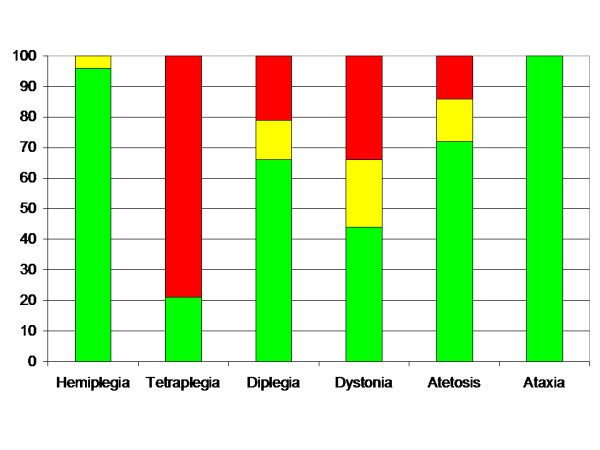
Proportion of children (%) with MP < 33% (green), 33–39% (yellow) and > 40% (red) in relation to subdiagnosis.

The distribution of MP related to subtype is presented in Table 7 and Figure 3. No child with spastic hemiplegia or pure ataxia developed MP > 40%. The highest risk of hip displacement was seen in children with spastic tetraplegia, where 11 of 14 children (79%) had MP > 40%. Children with spastic diplegia and the dyskinetic forms showed intermediate risk of hip displacement.

**Table 7:** Distribution of children with lateral hip displacement above different levels in relation to CP-subtype and GMFCS-level in 212 children with CP.

**CP-subtype/GMFCS-level**	**MP < 33%**	**MP 33–39%**	**MP > 40%**	**Total**
**Spastic**				
Hemiplegia	68	3	0	71
Tetraplegia	3	0	11	14
Diplegia	61	12	19	92
**Dyskinetic**				
Dystonia	8	4	6	18
Atetosis	5	1	1	7
**Ataxic**	9	0	0	9
**Unclassified**	1	0	0	1
				
**GMFCS**				
I	98	5	0	103
II	26	1	3	30
III	11	7	4	22
IV	11	5	13	29
V	9	1	18	28

**Figure 4:**
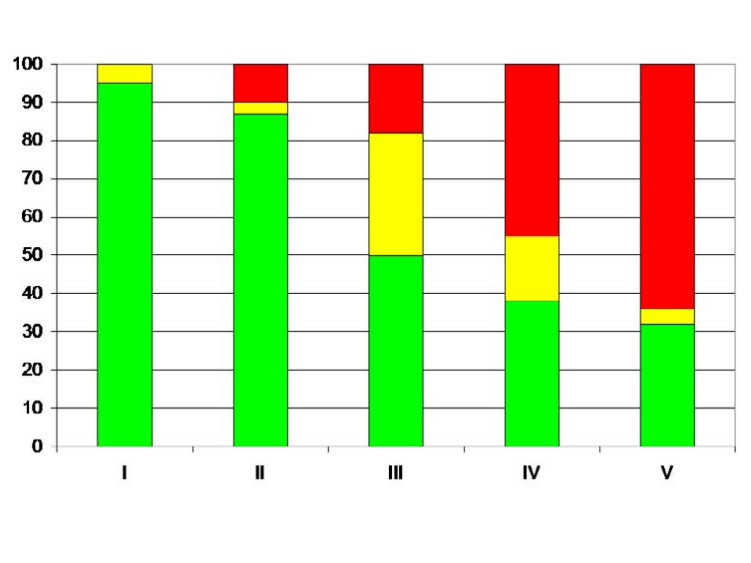
Proportion of children (%) with MP < 33% (green), 33–39% (yellow) and > 40% (red) in relation to GMFCS level.

The risk of hip displacement increased in relation to the level of gross motor function according to GMFCS (Table 7, Figure 4). No child with the highest level of function, GMFCS I, developed MP > 40%, while 18 of 28 children (64%) with the lowest level of function, GMFCS V, developed MP > 40%.

## Discussion

This is a population based study of 212 children with CP followed until 9–16 years of age. Most of the children have not completed growth. However, it is known that most dislocations in children with CP occur before seven years of age [15]. This is also supported by the decreasing number of children with debut of displacement after 3 years of age in the present study (Figure 2).

Lateral displacement of the femoral head is common without acetabular dysplasia and acetabular dysplasia develops at a later stage than femoral head lateralisation [16]. Measurement of the lateral displacement is sufficient for hip screening in CP [7,13]. The MP is a valid and reliable measure of lateral displacement [13,17], and probably the most commonly used. We choose the two levels of MP, 33 and 40% in the follow-up programme, CPUP, and in this study. When introduced, a hip with MP > 33% was the definition of a subluxated hip [18]. Some hips with MP 33–40% do not need preventive surgery, and these hips should be regarded as hips at risk, needing intensified observation, and operative intervention if increasing MP is seen. Hips with MP ≥ 40% have a high risk of further displacement, indicating need for surgical intervention [16]. The surgery performed in this materal has been described earlier [6].

The most common age at first registration of MP > 33% or > 40% was 3–4 years (Figure 2). Some of the children showed displacement at the first radiographic examination in the follow-up, so the real age at development of lateral displacement could be even lower. Some children showed lateral displacement already at two years of age. This means that it is of the utmost importance that children with CP are identified early, and that children at risk are examined radiographically as early as possible. Scrutton and Baird [19] recommended that children with spastic diplegia or quadriplegia should have a first radiograph at age 30 months. Based on our findings we recommend that children with highest risk should have their first radiographic examination even earlier, if possible.

Hip displacement in relation to CP-subtype could be divided into three levels of risk. Children with spastic hemiplegia or ataxia had very low risk, children with spastic diplegia or the dyskinetic types (dystonia and atetosis) had an intermediate risk, and children with spastic tetraplegia had the highest risk (Figure 3). There are, however, several problems using CP-subtype as indicator of hips at risk in screening programme. It is sometimes difficult to distinguish spastic hemiplegia from spastic diplegia with asymmetric severity, or pure ataxia from ataxic diplegia. The descriptors for the motor types of CP have not been universally agreed on; new definitions are under development, and the definitions may be difficult to apply in a reliable manner [20]. It is sometimes impossible to determine the CP-subtype before 4 years of age.

Hip displacement was directly related to the level of GMFCS (Figure 4). Similar results have been shown from the Victorian Cerebral palsy register [21]. The GMFCS has proved to be a valid and reliable tool [12] and has been reported to remain relatively stable over time [22,23]. According to the designers of the GMFCS, most children will remain at the same level from age 2 [24]. By using GMFCS, instead of CP-subtype, as an indicator of hips at risk in screening programme, all the problems discussed with subtypes seem to be avoided.

When the CPUP screening programme was initiated in 1994, the GMFCS was not available. Based on the findings in this and earlier studies we now have changed our programme for radiographic hip screening, and use GMFCS instead of subdiagnosis (Table 8). Most children with spastic hemiplegia and ataxia are at level GMFCS I, and all children with spastic tetraplegia are at level V. Children with spastic diplegia represent about one third of the population of children with CP and are distributed at all levels of function according to GMFCS. By changing to the new screening programme, those with spastic diplegia and GMFCS level I or II no longer need yearly radiographic examinations. The new screening programme results in about 35% fewer radiographic examinations up to 8 years of age in a total population of children with CP, compared with our previous programme.

**Table 8:** The radiographic prevention programme 2007

GMFCS I	No radiographic examination.
GMFCS II	Radiographic examination at 2 and 6 years of age*.
GMFCS III-V	Radiographic examination at diagnosis, then at least once a year until 8 years of age, then individually.
Children with pure ataxia	No radiographic examination.

* Further radiographic examination if MP > 33%, or a if a decreasing hip-ROM is revealed in the CPUP

The increased risk of hip displacement related to range of motion found in the crude estimates (Table 6) disappeared in the adjusted estimates. The increased risk described is therefore likely to be influenced by either the distribution of age, GMFCS-level or CP-subtype in the study population. The narrow confidence intervals corresponding to the adjusted estimates indicate that no change in risk seems to follow an increase in either measurement of ROM. This implies that the measurement of the range of motion is a poor indicator of risk and cannot replace radiographic examinations for hip screening. However, a decreasing ROM over time in an individual child could warrant radiographic hip examination.

## Conclusion

Hip displacement in CP often occurs already at 2–3 years of age. For a successful screening programme, it is of the utmost importance that children with CP are identified early, and those at risk of hip dislocation are examined radiographically as early as possible. Hip ROM is a poor indicator of risk, and not useful for screening. We recommend using the GMFCS system for construction of the screening programme.

## Competing interests

The author(s) declare that they have no competing interests.

## Authors' contributions

GH and HLP designed the study. All three authors analysed the results. GH wrote the first draft, which was then actively improved and revised by all three authors.

## Pre-publication history

The pre-publication history for this paper can be accessed here:


